# Assessing Physical Therapists’ Expectations and Experiences With an Automated Rehabilitation System Using Technology Acceptance Model: Multiple Methods Pilot Study

**DOI:** 10.2196/67440

**Published:** 2025-08-07

**Authors:** Cynthia Williams, Lindsay Toth, Raine Osborne, Chloe E Bailey, Aishwarya Joshi

**Affiliations:** 1 School of Global Health Management and Informatics, College of Community Innovation and Education, University of Central Florida, 528 W. Livingston Street Ste 401, Orlando, FL, 32081, United States, 1 3212766287; 2 Department of Clinical and Applied Movement Sciences, Brooks College of Health, University of North Florida, Jacksonville, FL, United States; 3 Department of Physical Therapy, Brooks College of Health, University of North Florida, Jacksonville, FL, United States; 4 Brooks Rehabilitation, Jacksonville, FL, United States

**Keywords:** wearable technology, rehabilitation, physical therapy, technology acceptance model, inertia movement units, wearables, mhealth, rehabilitation medicine, technology use, survey, mobile health

## Abstract

**Background:**

Wearable sensor systems maximize visual and clinical feedback for physical therapists to enhance patient outcomes in rehabilitation medicine. However, physical therapists must adopt and accept new technologies for full integration into routine care to advance the use of technology in clinical care. Their role in technology design is critical in adopting and implementing technology. Interprofessional collaboration should be supported in the design of rehabilitation-assisted technologies.

**Objective:**

We used the established tenets of the Technology Acceptance Model to describe physical therapists’ expectations and experiences before and after using a novel wearable system in outpatient physical therapy.

**Methods:**

This multiple methods pilot study used a comparative pre-post survey and a qualitative semistructured focus group study design. Using purposive sampling, we recruited outpatient physical therapists to pilot the novel wearable technology, describe their expectations and experiences, and participate in a semistructured focus group discussion conducted to gather training and user experience information.

**Results:**

The study sample consisted of 5 physical therapists with an average age of 38.8 (SD 6.9) years and a work experience average of 12 (SD 7.7) years. Presurvey data show favorable expectations for usefulness and ease of use; however, favorability in both factors decreased after use. For perceived usefulness, all responses moved in the less favorable direction; mean difference −4.4 (SD 3.21); *P*=.04. All but 2 responses moved in the less favorable direction for overall perceived ease of use; mean difference −4.8 (SD 1.79); *P*=.04. Themed responses to open-ended questions in the postsurvey were feedback, setup time, accuracy, performance, and enhanced functional activities. Inductive content analysis of the focus group responses resulted in the following themes: system training, system benefits, system challenges, physical therapist perception of patients, and suggestions for improvement. The expectation for frequency of use decreased pre- to postexperience by 53% (mean −22, SD 14.40; *P*=.04).

**Conclusions:**

The Technology Acceptance Model–based survey responses and focus group themes outcomes demonstrated that physical therapists’ expectations for using new technology were not met. Engaging physical therapists in piloting novel wearable technology highlights the importance of physical therapist engagement in developing, refining, and implementing wearable devices for rehabilitation.

## Introduction

Advances in wearable sensor technology have accelerated the use and reliance on digital biomarkers in clinical settings to enhance patient care outcomes. In rehabilitation medicine, wearable sensor systems provide new opportunities for collecting, storing, analyzing, and visualizing movement-related data [1,2]. In addition, wearable technology enhances opportunities for real-time information to support clinical decision-making and promote treatment progress. However, the speed of innovative development and other factors, such as usability, need, trust in technology, and cost often outpace or otherwise interact with adoption [3].

In rehabilitation medicine, engaging the end users (ie, physical therapists) in piloting novel technology can enhance usability and adoption so that design and output requirements meet expectations [4]. Such partnerships can assist technology developers and engineers in the product development and refinement stage [5]. A wide range of criteria have been suggested for consideration in adopting new rehabilitative technology, including observed criteria, such as patient and provider use, positive clinical outcomes, and fiscal viability [6,7]. However, to optimize behavioral investigation, a widely accepted framework should be used in pilot testing to garner user feedback. This is critical for gaining an understanding of the acceptance and adoption of new technology before full implementation [8].

Based on the theory of reasoned action [9] and the theory of planned behavior [10], the Technology Acceptance Model (TAM) offers a parsimonious explanation of individuals’ behavior toward technology acceptance [6,11]. Although physical therapists’ satisfaction is a well-accepted factor in technology acceptance, behavioral intention to use technology is one of the most fundamental antecedents to technology usage [1]. The behavioral intention to use was shown to be impacted by 2 primary beliefs: perceived usefulness and perceived ease of use [3,6,11]. Perceived usefulness is the extent to which physical therapists believe that using a specific system will improve their job performance (eg, time and case management, clinical decision-making, and patient outcomes), while perceived ease of use is the extent to which they believe using the system will be effortless [6,12]. When technology is easy to use, it fosters a positive attitude, leading to greater use [3]. Thus, perceived ease of use is directly associated with perceived usefulness, and both concepts impact physical therapists’ intentions to adopt technology, considering external factors, namely, the implementation process, user participation, training, and system characteristics [6,11,12].

Several reviews have shown that the TAM is validated in health care settings. In studies focused on clinicians’ adoption of health-related technology, this model repeatedly explains substantial variance in the intention to use or actual use of health technologies [1,13-15]. Furthermore, this model is accepted as a method of investigating novel technology in outpatient physical therapy [16,17]. Given its accepted use in outpatient physical therapy and its predictive ability, this study used the established theoretical framework to describe physical therapists’ expectations and experiences before and after using a novel wearable system in outpatient physical therapy.

The knee joint represents a significant area of interest, given the prevalence of knee injuries across populations that require conservative and postsurgical management [18]. While wearable technologies are often used in orthopedics for their diagnostic capabilities, there is less research on knee rehabilitative technologies for everyday clinical practice [18]. Thus, this study will add to the literature on the practical use of wearable technology in clinical settings as an adjunct to medically necessary knee rehabilitation.

The study builds on previous research indicating that a clinical application for the automated rehabilitation system is needed [11] and explores the question “What are physical therapists’ perceptions regarding the design and functional requirements of innovative wearable technology for knee rehabilitation?” The goal is to examine physical therapists’ views on knee wearable technology within a clinical setting, using their expertise to guide the system’s adoption and implementation. This study establishes a framework for incorporating physical therapists’ feedback into the technology design process, aiming to enhance factors that promote technology acceptance in everyday clinical practice.

## Methods

### Study Design and Participants

This pilot study used a multiple-methods design, a comparative pre-post survey, and a qualitative semistructured focus group. We used purposive sampling to recruit full-time physical therapists providing direct care services in outpatient clinics from 1 outpatient rehabilitation organization with several clinics throughout Northeast Florida. A research team member recruited physical therapists after administrative approval from participating outpatient physical therapy clinics.

### Automated Rehabilitation System

The automated rehabilitation system (ARS) is a prototype wearable sensor system that uses inertial measurement unit (IMU) sensors and ARS software on a tablet to collect, store, and visualize knee motion in real-time. The IMU sensors are enclosed in small, lightweight plastic casings (3.8 cm×3.8 cm×1.9cm) and affixed to the patient’s skin using kinesiology tape at specific anatomical landmarks (anterior leg, 4 inches above and below the patella on each leg). Once affixed to the skin and synchronized to the software, the sensors transmit accelerometer and gyroscope time series data to the ARS software. This software applies pose estimation and time-series segmentation algorithms to estimate knee angles and velocity, facilitating data collection, storage, and visualization. The technical details about this system are published elsewhere [19,20].

**Figure 1.**
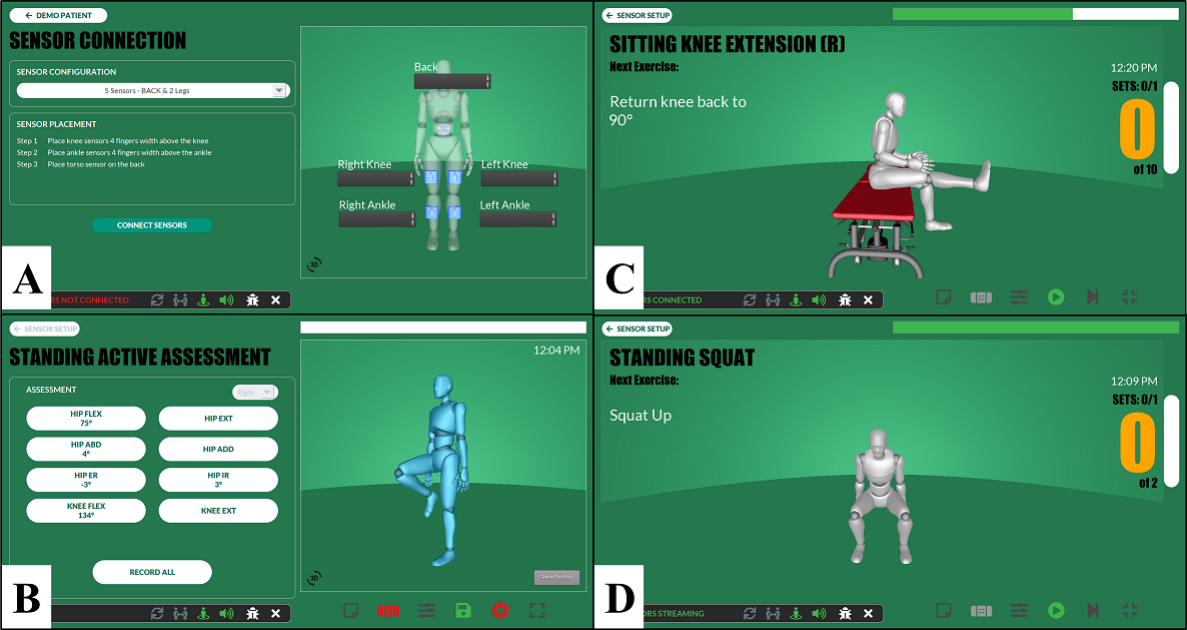
Graphical user interface for the automated rehabilitation system.

The ARS software provides 2 graphical user interfaces (GUIs), one for physical therapists and one for patients, on the associated tablet (Figure 1). The clinician’s GUI allows the creation of patient user accounts, includes step-by-step instructions for completing and storing range of motion (ROM) assessments, and provides an area for creating exercise programs and progressions for each patient by selecting exercises from an exercise library. The clinician’s GUI also includes summary reports of completed exercises, the number of complete repetitions, knee ROM, and velocities for each exercise and repetition. The patient’s GUI displays an avatar showing the patient’s movement in real-time. The avatar guides the patient through the full ROM for each exercise and counts each complete repetition. The ARS is designed to improve exercise adherence, effectiveness, and care continuity while creating a more engaging experience for physical therapists and patients [19,20].

### Protocol

After completing the electronic informed consent via Qualtrics (Silver Lake Technology Management, LLC) [21] and before exposure to the ARS, physical therapists completed a questionnaire (presurvey) of demographics and 17 items related to general expectations for the use of wearable sensor technology in clinical practice. The TAM was used as a framework for the survey, with 10 items addressing usefulness, 6 items addressing ease of use, and 1 item addressing frequency of use. The specific items representing usefulness and ease of use were developed through collaboration between research team members with clinical expertise and the system engineers. Items were designed to assess aspects of usefulness and ease of use that would inform the development and clinical adoption of wearable sensor technology. In addition to these items, physical therapists were also asked to rate the overall perception of usefulness and ease of use. Perceived usefulness and perceived ease of use were conceptualized as bipolar constructs. Items related to these constructs were scored on 21-point, bipolar slider scales with a neutral-centered starting point, such that physical therapists had to decide on which side of neutral their response would fall (directionality) and the level (magnitude) of their response [22,23]. Physical therapists indicated the magnitude of their positive or negative expectations using the anchors −10=not at all useful, 0=neutral, 10=extremely useful for perceived usefulness, and −10=extremely difficult, 0=neutral, 10=extremely easy for perceived ease of use.

The frequency of use item represents a modification of the intention to use construct from the TAM [1]. While the intention to use assesses a physical therapist’s expectation to use or not use a technology, the frequency of use item was intended to assess how often a physical therapist expects to use the technology. This distinction was deemed relevant and important by the research team and system engineers to help determine the degree to which physical therapists expect to use wearable sensor technology in clinical practice with a wide variety of patients, selectively with a few patients, or not at all. For consistency, frequency of use was also measured on a 21-point scale using the anchors (−10=none of my patients, 0=about half of my patients, and 10=all of my patients). The physical therapists were also given 6 potential use cases for wearable sensor technology (eg, at home during a home exercise program) and asked to rank the use cases in order from most to least beneficial uses for wearable sensor systems.

After completing the presurvey, each physical therapist was required to attend a 2-hour in-person ARS training session provided by the research team and a member of the technology development team responsible for the GUI design. The training session included practice affixing the IMU devices around the knee, initial patient profile creation with the ARS software, using the software to complete ROM assessments, and designing exercise programs. The training concluded with a mock patient trial with 2 case studies. The physical therapists were asked to demonstrate appropriate setup and use procedures before leaving the training session. After the session, physical therapists were provided with an ARS and were asked to practice with the technology for a 1-month trial period. During the trial period, physical therapists were encouraged to contact a research team member for technical support and assistance. Any technical issues that a research team member could not resolve were communicated to the system engineers either by the research team member or the physical therapist for problem-solving. After the trial period, the physical therapists began applying the ARS in their physical therapy practice with qualified patients.

Physical therapists were instructed to use the system during outpatient in-clinic visits with patients who meet the inclusion criteria: patients who meet the inclusion criteria of being at least 18 years of age, seeking physical therapist services for a diagnosis related to an orthopedic knee condition (new patient or existing patient with new knee-related diagnosis) and having no cognition-related diagnoses. Physical therapists used the ARS during routine clinical assessments and treatments (on average 4‐6 wk, 8‐12 sessions, in-clinic only) with patients who consented to participate in the study. Physical therapists were not required to follow a specific protocol but were instructed to administer medically necessary care and use the device where appropriate during the entire episode of care. The research team and system engineers provided ongoing technical and procedural support throughout the study.

After using the device with 5 patients who completed their episode of care, the physical therapists were asked to complete a postsurvey with items related to their experience using the ARS. The survey included 17 items similar to the presurvey, with wording slightly modified to ask about experience instead of expectation. The postsurvey included 6 additional open-ended items that asked physical therapists to describe their experience using the ARS, such as situations where the ARS was most useful, weaknesses of the ARS, and recommendations for further development or modifications.

After completing the survey, the physical therapists participated in a focus group discussion via Zoom video conference (Zoom Communications) [24]. The one-and-a-half-hour focus group was held with all physical therapists and moderated by 2 research team members (CW and RO). A semistructured interview guided the discussion and included questions addressing training, experience, and perceived patient experience. The questions (Table 1) are intended to provide a richer understanding of the physical therapists’ survey responses and perspectives. They were designed to promote free-flowing conversation and elicit frank discussions about their experiences with technology. Follow-up questions were asked to clarify or explain comments.

At the conclusion of the study, members of the research and technology development teams met to discuss the identified results of the surveys and interviews. This discussion provided additional context to the findings and potential modifications to the system and clinical training procedures that may inform future research and practice.

**Table 1.** Focus group questions.

Training and experience	Initial questions	Follow-up questions
Training	Tell us about your experience learning how to use wearable sensors.	What went well, and what could have gone better?
Experience	Tell us about your initial experience using wearable sensors with patients.	How did this change over time? Please elaborate on the technical challenges. Discuss the exercises you would like to see in the system. The group mentioned visual feedback. How do you think this influenced the therapy session?

### Statistical Analysis

The survey data analyses were performed using IBM SPSS Statistics (version 29.0). The pre- and postmean rank and SD were also calculated for the use case rankings. Due to low sample size and non-normal data distributions, the Wilcoxon Signed Rank test was used to assess statistically significant differences between pre- and postscores and rankings.

### Qualitative Analysis

The focus group session was audio recorded and transcribed verbatim using the Zoom recording function [24]. The focus group included researchers and physical therapists; no nonparticipants were included. The transcribed responses were validated with the audio recorder for accuracy and deidentified for analysis. An inductive content analysis approach was used to examine and interpret the qualitative data. The analysis used an inductive bottom-up approach and semantic coding to identify explicitly stated ideas, concepts, and experiences. The process began with a thorough reading and rereading of the paper to increase familiarity with the content and understand the concepts in each response. Then, initial codes were generated through line-by-line coding of the transcripts. After this, subthemes were developed by clustering overlapping codes and reanalyzing them to ensure alignment with the responses. After reviewing and clustering the codes, the authors searched for subthemes that could be combined to reduce redundancy and highlight salient patterns in the data. Following this phase, 2 authors (CW and RO) discussed the subthemes, and discrepancies were resolved by a third researcher (LT). This refinement led to the identification of overarching themes. The process was continuously revised, refined, and discussed among the research team. We used the consolidated criteria for reporting qualitative research checklist to ensure explicit reporting on the study’s execution [25].

### Ethical Considerations

Physical therapists who verbally agreed to participate in the study received and signed the electronic informed consent before beginning the study protocol. This study followed the research protocol approved by the University of North Florida’s Institutional Review Board (approval number 21‐041 1710770).

## Results

### Participants

A total of 5 physical therapists aged 31-50 years with 4-25 years of practice experience consented to participate in the study. The study sample reflected an average age of 38.8 (SD 6.9) years and a work experience average of 12 (SD 7.7) years. All reported being at least somewhat comfortable with technology (Table 2).

**Table 2.** Description of participating physical therapists.

Characteristics	Clinician 1	Clinician 2	Clinician 3	Clinician 4	Clinician 5
Age (y)	31	50	41	40	32
Sex	Female	Male	Male	Female	Female
Years of physical therapy experience (y)	4	25	10	16	5
Highest degree in physical therapy	DPT	BS	DPT	DPT	DPT
Comfort level with technology	Somewhat Comfortable	Comfortable	Comfortable	Somewhat Comfortable	Comfortable

### Survey Results

Pre- and postsurvey results highlight the perceptions of the therapist before and after using the technology in patient care. Table 3 highlights the pre- and postresults and mean differences according to the TAM. The physical therapists’ expected frequency of using the device with patients significantly decreased from pre- to postuse of the wearable system, with a mean difference of −22 (SD 14.40; *P*<.05).

**Table 3.** Clinicians’ perceived usefulness and ease of use pre and post use of the wearable sensor system.

Constructs	Pre, mean (SD)	Post, mean (SD)	Difference, mean (SD)		*P* value
Usefulness					
Improving patient outcomes	4.4 (3.29)	−0.6 (4.98)	−5.0 (5.92)		.14
Increasing the speed of recovery	0.8 (3.03)	−1.4 (3.58)	−2.2 (4.38)		.29
Improving the quality of care I am able to provide to patients	4.0 (3.24)	0.2 (4.82)	−3.8 (5.45)		.22
Improving the cost-effectiveness of rehabilitation services	0.4 (3.21)	−0.6 (4.34)	−1.0 (5.87)		.85
Increase my patients’ satisfaction with their care	5.2 (3.63)	0.8 (3.96)	−4.4 (4.04)		.07
Increase my level of efficiency in the clinic	0.0 (3.74)	−4.4 (2.19)	−4.4 (4.77)		.10
Providing me with valid and reliable information about patients	3.8 (2.05)	−1.2 (3.96)	−5.0 (2.83)		.04^a^
Improving my clinical decision-making	1.2 (1.30)	−2.0 (2.12)	−3.2 (2.39)		.07
Improve my ability to monitor patient progress	4.0 (1.41)	−1.2 (3.11)	−5.2 (3.11)		.04^a^
Overall perceived usefulness	3.8 (1.79)	−0.6 (2.97)	−4.4 (3.21)		.04^a^
Ease of use					
Learn how to use wearable sensor technology	6.2 (2.28)	4.4 (4.04)	−1.8 (4.09)		.41
Incorporate wearable sensors into my usual practice patterns	0.4 (1.82)	0.6 (2.97)	0.2 (3.96)		>.99
Explain wearable sensor technology to patients (purpose and benefits)	5.6 (2.97)	6.4 (5.32)	0.8 (3.56)		.72
Teach patient how to use wearable sensor technology	4.8 (3.11)	4.4 (3.78)	−0.4 (2.97)		.79
Maintain the equipment associated with wearable sensor technology	6.2 (3.90)	6.0 (5.66)	−0.2 (5.76)		>.99
Overall perceived ease of use	1.4 (1.95)	−3.4 (1.52)	−4.8 (1.79)		.04^a^
Frequency of use					
I would use wearable sensors with	47 (15.65)	25 (12.75)	−22(14.40)		.04^a^

a P≤.05; usefulness scale (−10=not at all useful, 0=neutral, 10=extremely useful); ease-of-use scale (−10=extremely difficult, 0=neutral, 10=extremely easy); frequency of use scale (0=none of my patients, 50=about half of my patients, 100=all of my patients); change reflects the post score minus the prescore such that negative values represent a decrease in perceived usefulness, ease-of-use, or frequency of use.

#### Perceived Usefulness

Presurvey results indicated that physical therapists had neutral to moderate positive expectations for the usefulness of wearable sensors in clinical practice, with the lowest expectations for “increasing my level of efficiency in the clinic” (mean 0, SD 3.4), “increasing the cost-effectiveness of rehabilitation services” (mean 0.04, SD 3.21) and “ increasing the speed of recovery” (mean 0.08, SD 3.03). The highest expectation was for “increasing patient satisfaction with their care” (mean 5.2, SD 3.63), “improving patient outcomes” (mean 4.4, SD 3.29), “improving the quality of care I am able to provide patients” (mean 4.0, SD 3.24), and “improving my ability to monitor patient progress” (mean 4.0, SD 1.41). The overall pre-exposure perceived usefulness mean was 3.8 (SD 1.79). Following exposure to the ARS, physical therapists’ overall perceived usefulness score was −0.6 (SD 2.97), representing a statistically significant decrease in the perceived overall usefulness of wearable sensors; *P*<.05; mean difference −4.4 (SD 3.21). Although pre- and postexposure mean difference decreased for all usefulness items, the only statistically significant (*P*<.05) observed decreases were in the items related to the ability to monitor patient progress (mean difference −5.2, SD 3.11) and the quality of the information from the system (mean difference −5.0, SD 2.83).

#### Perceived Ease of Use

Physical therapists expressed pre-exposure perceptions that it would be relatively easy to “learn how to use wearable sensor technology” (mean 6.2, SD 2.28) and “maintain the equipment associated with wearable sensor technology” (mean 6.2, SD 3.90). Following exposure to the ARS, physical therapists’ overall perceived ease of use score was −3.4 (SD 1.52), representing a statistically significant decrease in the perceived overall ease of use of wearable sensors *(P*<.05; mean difference −4.4, SD 3.21). No statistical changes were seen in individual questions with ease-of-use perceptions.

#### Use Cases

Physical therapists’ ranking of the use cases remained largely unchanged between the pre- and postsurvey, with “At home during a home exercise program” consistently ranked as the most beneficial and “other” consistently ranked as receiving the lowest rank. Although in sport/recreation settings, the ranking moved from fifth-ranked in the presurvey to fourth-ranked in the postsurvey, there were no statistically significant differences observed in the rankings (Table 4).

**Table 4.** Clinicians’ ranking of most beneficial uses for the wearable sensor system.

Potential Settings for ARS Use	Pre, mean (SD)	Post, mean (SD)	Difference, mean (SD)	*P* value^a^
At home during a home exercise program	2.0 (1.73)	1.0 (0.00)	1.0 (1.73)	.18
In the clinical setting as part of rehabilitation services	2.4 (1.14)	2.6 (1.34)	−0.2 (0.84)	.56
At home during usual daily activities	2.8 (1.10)	3.4 (0.55)	−0.6 (1.14)	.26
At work/school	4.4 (0.55)	3.6 (1.14)	0.8 (1.48)	.26
In sports/recreational settings	3.4 (1.52)	4.4 (0.89)	−1.0 (2.12)	.27
Other	6.0 (0.00)	6.0 (0.00)	0.0 (0.00)	>.99

a *P*≤.05; Clinicians ranked use cases for the wearable sensor system in order from most beneficial (1) to least beneficial (6). Change reflectst pre score minus post score so that negative values represent a decrease in ranking.

#### Clinical Comments

Physical therapists were asked 5 open-ended questions at the end of the postsurvey. These responses highlighted favorable perceptions and areas where improvements could be made. For example, the physical therapists highlighted where wearable technology could be beneficial by providing visual feedback; the visual feedback would support the correct performance of exercises. They also expressed that they like the ability to assess multiple joints simultaneously. They commented that the device would be suitable for home exercise programs and would enhance their in-clinic ability to evaluate range of motion (ROM). However, the setup time and calibration requirements were barriers to use. In addition, they recommended that designers improve the ability to don and doff the sensors, improve the accuracy of the sensor’s feedback, and provide more functional activities to expand the system’s capabilities. Table 5 shows a summary of the comments by question.

**Table 5.** Summary of post-survey open-ended questions.

Questions	Themes
What are one or two examples of a situation where you found using the wearable sensor system to be most useful?	Visual feedback Consistency in supporting correct performance Measure multiple joints at the same time
What are one or two of the features/aspects of the wearable sensor system that you especially liked?	Good for home exercise program Assess ROM^a^ Visual feedback
What are one or two of the biggest weaknesses in the wearable sensor system?	Set up time and calibration Inaccuracy of feedback
What are one or two things you would recommend for further development of the wearable sensor system?	Easier to don and doff Improve accuracy
Can you think of any other ways you might want to use a wearable sensor system?	More functional activities
What would have been useful to know during the training that you were not told?	N/A^b^

a ROM: range of motion.

b N/A: Not applicable.

### Focus Group

**Figure 2.**
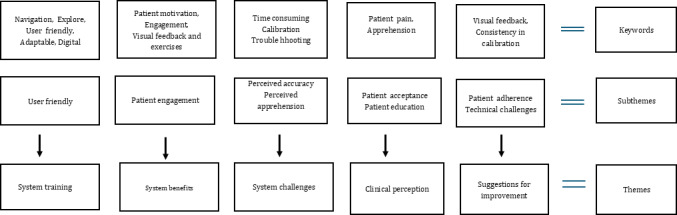
Summary of keywords to themes in qualitative analysis.

The qualitative results from the focus group were organized by question: training and experience using the device. Within each question, the comments were organized by keywords, subthemes, and themes. Following the inductive content analysis, the focus group revealed 4 themes: system training, benefits, challenges, physical therapists’ perception of patient experience, and suggestions for improvement. The themes were then evaluated against perceived ease of use and perceived usefulness. Please refer to Figure 2 for the keywords, subthemes, and theme development.

#### System Training

In the theme of system training, physical therapists expressed positive feedback about the required training session on using the device.

*So I think the training was perfectly fine. I think the system as a whole is pretty user friendly, and as long as you kinda take the time to like, explore and kind of play around with adding new exercises, or deleting or changing [repetitions], or anything like that it’s pretty easy to use.*

[PT-4]

Another physical therapist agreed that the training was good; however, they commented that being familiar with technology was helpful.

*I mean, I think it’s going. I think the training that we did was good. I mean it was I didn’t have any issues with it. I don’t know. Technological, savvy? It helped it.*

[PT-5]

#### System Benefits

In the theme of system benefits, physical therapists commented that the system’s visual feedback motivated the patients during rehabilitation.

*So it’s almost kind of made them push themselves a little bit more. I think a lot of the patients like the fact that it’s counting their reps for them.*

[PT-4]

*Yes, I’d agreed with that. and I’m a big fan of like bio feedback. I think when the thing is working and counting. I think that’s a huge plus for the patients to go through, and, you know, have something to compare their movement to that.*

[PT-3]

#### System Challenges

In the theme of system challenges, the subthemes of perceived accuracy and perceived patient apprehension were also noted. Physical therapists expressed technical challenges due to calibration issues that led to reservations about the accuracy of the data.

*I think probably half the time, I would say I’d have to spend troubleshooting not half of the session time, but half of the occasions that I was using to sensors. I would have to spend some extra time troubleshooting. So I think I kind of had a few random occurrences of that where we would maybe transition from like supine mat exercises to just sitting or standing.*

[PT-4]

*That’s definitely a challenge. So the time that you’re having to fight with recalibrating. It’s just when it starts having a difficulty tracking. are you confident in the data that it provides you?*

[PT-1]

*Yeah, I'd agree. The biggest barrier for me using it is just trusting that the data that it’s giving me is accurate.*

[PT-2]

However, they were confident in the accuracy when it was working well.

*As far as the watching them doing their exercise is seeing the range that is going through all that stuff. I’m pretty confident. With that I went through, and I’d kind of measured range of motion and I measured range of motion to see how close it was. So. Yeah. I was pretty comfortable with that. It’s just when it starts having a difficulty tracking.*

[PT-2]

The physical therapists also perceived that those technical challenges impacted the patients’ perceptions of the technology. When the system had difficulty, they were aware of patient time and apprehension. One physical therapist noted:

*And all of a sudden, you know he’s not kind of displaying the correct position. and so from there I would pretty much just kind of like, say, okay, if we can’t recalibrate this and get it to appear correctly within the next slide. 2 min or so. We’ll just go ahead and continue with the exercise, and you know, not worry about it.*

[PT-3]

#### Clinician Perception of the Device

The physical therapists’ perception of the device also reflected their perception of the patients’ experience. Thus, the physical therapists’ perception of the device had several subthemes: patient acceptance and patient education. On the subtheme of patient acceptance, physical therapists perceived that patients’ interest may be related to their level of pain.

*I mean for a lot of people, especially like when you get the patients to come in with like a the [knee] replacement or something like that, or they’re coming in a lot of pain. They’re probably coming in a week, or, you know, within the first week after surgery, and it’s kind of like they what they don’t want anything else. They’ve got enough going on. They don’t want anything else on top of it.*

[PT-3]

Another physical therapist suggested that more patient education was needed to gain patient acceptance of the technology.

*So maybe, having the clinicians like talk to the patient and explain it’s not going to change anything we’re doing with them, and all that kind of stuff would help but just to kind of give their mind that it’s not going to be a big hindrance to them. But I’ve definitely had an issue with a lot of people not want[ing] to do it.*

[PT-4]

#### Suggestions for Improvement

Physical therapists commented on the challenges of the system. They also made recommendations in the subthemes of patient adherence and technical challenges to improve patient adherence throughout their treatment program.

*The things that we’re doing are good for patients early on or for really low-level patients. But eventually, for a lot of patients, they’re going to get beyond what we’re doing at this time.*

[PT-5]

While being able to track functional movements visually was important for patient care, the therapist suggested that improving the challenges with calibration was also critical to improving the use of the system.

*Being able to tracking and functional activities. It’s probably the biggest improvement, and then just the consistency of the calibration that that would be the biggest issue that I run into.*

[PT-1]

## Discussion

### Principal Findings

Implementing wearable technology into clinical practice is a significant step forward in rehabilitation science. The ubiquitous nature of wearable technology to monitor health behaviors and improve physical performance, and the lack of in-clinic rehabilitation use, suggests that rehabilitation medicine should consider technology that could effectively promote practice. This study exposed a sample of orthopedic outpatient physical therapists to a new, premarket wearable system to garner their perceptions about the device for possible implementation into clinical practice. The survey and focus group results indicate that additional work is necessary before the studied prototype can fully integrate into clinical practice. These findings underscore the participating physical therapists’ expectations, perceptions, and recommendations. In addition, the study emphasizes the significance of interprofessional collaboration in developing technology for clinical care. Our analysis indicates that while physical therapists were enthusiastic about the system, its perceived usefulness and ease of use did not fully meet their expectations. These perceptions were influenced by physical therapists’ observations of patients’ responses during rehabilitation sessions and their own experiences with the device. As a result, technical challenges reduced the overall perceived usefulness, ease of use, and frequency of use. The focus group offered suggestions to enhance the device’s acceptability in clinical practice, as highlighted by the theme of suggestions for improvement, which aligns with the theme of system challenge.

### Perceived Usefulness

Perceived usefulness, as the TAM suggests, is critical for technology adoption. In this study, survey results indicate a significant decline in the perceived usefulness of the wearable system from pre- to postsurvey. Negative perceptions were primarily influenced by doubts about the system’s ability to provide valid and reliable information and monitor patient progress, as highlighted by specific survey questions. These findings were corroborated by qualitative data from focus group themes of system challenges and suggestions for improvement. From the focus group-generated theme of system challenges, subtheme of perceived accuracy, the physical therapists expressed technical issues, such as lagged visual feedback and calibration problems, which led them to question the accuracy of the data and hindered the system’s usability. Consequently, troubleshooting these issues interrupted patient care. This is an important issue, as Argent et al [26] indicated that data reliability and accuracy are major challenges in accepting new wearable technology in clinical practice. Under the theme of system challenges, the subtheme perceived apprehension, technical issues causing delayed visual feedback can undermine the system’s effectiveness and erode the trust of both clinicians and patients. This, in turn, heightens the physical therapist’s reluctance to use the device. Health practitioners felt legally responsible for any adverse consequences resulting from potential inaccuracies or delays [27,28].

In the theme of suggestions for improvement, the subtheme of patient adherence contributed to the perceived usefulness of the wearable device. The physical therapists noted that the limited availability of exercises restricted the use of the ARS as patients advanced in their rehabilitation. The wearable system included basic knee rehabilitation exercises, but the therapists recommended that more advanced exercises were needed to optimize the system’s use. As patients progress in knee rehabilitation, they outgrow the exercises available in the system. This may contribute to survey results from physical therapists that the system might be more suitable for home exercise programs rather than clinical settings.

Conversely, in the theme of system benefits from the focus group discussion, physical therapists found the avatar valuable for patient engagement, noting that real-time visual cues could encourage patients to move through their full ROM. This supports the idea that real-time feedback is important for the effectiveness of wearable technology [28,29]. Visual feedback enhances motor learning outcomes beyond traditional physical therapy , allowing patients to see the correct movements and full ROM while responding to visual stimuli and the clinician’s vocal cues [30]. In addition, physical therapists favored visual feedback because it improved patients’ understanding of prescribed exercises and the accuracy of gross motor activities [31].

### Perceived Ease of Use

Perceived ease of use, alongside perceived usefulness, is an important factor in technology adoption. Integrating new technology into clinical practice should support rehabilitation; however, additional time and effort can hinder care delivery, making the system more challenging to use. Survey results indicate a significant decrease in ease of use during the study. This finding is corroborated by focus group results, which highlight the themes of system challenges, clinical perceptions, and suggestions for improvement. Furthermore, under the theme of system challenges (subtheme perceived apprehension) and suggestions for improvement (subtheme technical challenges), it was noted that patients felt uncomfortable with the time required for calibration and troubleshooting. Physical therapists were concerned that it was too time-consuming and distracted from the rehabilitation session. Patients required reassurance that their treatment would not change based on ARS [32,33]. In addition, within the theme of clinical perception, the subtheme of patient acceptance, pain, adversely affected the device’s ease of use. This suggests that the complexity of the diagnosis and the patients’ pain levels significantly influence their perception of wearable technology [34]. Patients with higher care needs might view monitoring with novel technology as unnecessary for their care. Some physical therapists also avoided using the technology, citing concerns that it would reduce patient contact time and the number of visits, negatively affecting care [35,36]. This study underscores a significant gap in the literature on technology adoption, emphasizing the importance of therapists’ perceptions of patients using wearables. Few studies illustrate therapists’ empathy for patients during rehabilitation with wearables [37,38].

### Implications for Practice

This study highlights the importance of interdisciplinary collaboration among engineers, physical therapists, and researchers to enhance wearable sensors’ design, output, and ultimate acceptability in rehabilitation. The physical therapists were initially open to the use of the ARS in clinical practice, but the challenges with the system were notable. The feedback from the physical therapists humbled the enthusiasm of technology engineers, but it provided them with valuable information to improve the prototype for potential physical therapists who may use the device in the future. System developers should be mindful of the concepts of TAM and physical therapists’ expectations for use in rehabilitation. Wearable technology developers are well-positioned, in a digitally aggressive health care climate, to be “digital disruptors” in traditional physical therapy settings; however, it cannot undermine well-established processes of care and the physical therapists-patients relationship [36]. Wearable technologies are being actively promoted to physical therapists by technology developers and corporations. However, technology must meet physical therapists’ demands for data validity and reliability [36,39]. Without meeting this minimum requirement, wearable technology could be seen as disruptive to patient care [40].

In the clinic, wearable monitors provide an opportunity for objectively measuring ROM and biomechanical assessment. Replacing goniometers with wearable sensor systems offers the benefit of digital ROM testing, where measurements could be compared with the patient’s previous results or population-based norms for assessing rehabilitation progress and benchmarks for returning to work or sports participation [18]. These systems also allow for portable, less expensive, and more intuitive biomechanical tracking of activities compared with current video and marker-based systems [41]. Integrating this technology may provide quicker detection of biomechanical errors and allow for earlier physical therapy intervention to mitigate issues.

### Future Research

Future collaboration should consider garnering physical therapists’ expectations before developing wearable technology. Due to the fiscal impact of technology adoption, studies should consider the financial factors of wearable systems. While studies suggest promising outcomes such as decreased costs associated with visits, reducing unnecessary clinic visits, reducing patients’ travel burden, and streamlining patient-clinician visits, more research is needed to quantify such results [42]. Other research postulates that a wearable rehabilitation sensor system would reduce time spent with physical therapists while instructing and tracking rehabilitation exercises [19]. This may increase clinician productivity while also promoting quality care. In addition, future research should overcome the sample size limitations and replicate the study during a period that is less likely to experience a public health crisis to promote more in-person visits.

### Limitations

The study should be considered in light of the limitations. The small sample size of physical therapists increases the risk of not detecting important postexposure differences and limits the generalizability to other settings and diagnoses. While the study presents a widely accepted framework, we cannot speculate on results in other rehabilitation clinics. In addition, the study period occurred during the COVID-19 pandemic and may not be reproducible as in-person patient care was limited. The COVID-19 pandemic negatively impacted the sample size. Future studies should consider a larger sample size across various settings with data that measure the intensity of use and consider other appropriate diagnoses for wearable devices in rehabilitation. While the study uses the basic tenets of the TAM, future studies may consider more complex versions of the TAM.

### Conclusions

This multiple-method study used the TAM to create a survey examining physical therapists’ expectations and experiences with a new wearable system in outpatient physical therapy before and after its use. The findings indicated a decline in perceived usefulness and overall ease of use among physical therapists. While the technology has the potential to enhance the efficiency of physical therapy care and monitor rehabilitation progress, careful attention must be given to the system’s design and accuracy during development. The feedback provided valuable insights for engineers and system developers to refine the system based on physical therapists’ perceptions. This study underscores the importance of involving physical therapists in the prototype development phase.

## Abbreviations

ARS: automated rehabilitation system
GUI: graphical user interface
IMU: inertial measurement unit
ROM: range of motion
TAM: Technology Acceptance Model

## References

[1] Holden RJ, Karsh BT (2010). The technology acceptance model: its past and its future in health care. J Biomed Inform.

[2] Motahari-Nezhad H, Fgaier M, Mahdi Abid M, Péntek M, Gulácsi L, Zrubka Z (2022). Digital biomarker-based studies: scoping review of systematic reviews. JMIR Mhealth Uhealth.

[3] Kim KJ, Shin D (2015). An acceptance model for smart watches: implications for the adoption of future wearable technology. Internet Res.

[4] Desplenter T, Chinchalkar S, Trejos AL Enhancing the therapist–device relationship: software requirements for digital collection and analysis of patient data.

[5] Nijboer F, Plass-Oude Bos D, Blokland Y, van Wijk R, Farquhar J (2014). Design requirements and potential target users for brain-computer interfaces – recommendations from rehabilitation professionals. Brain-Computer Interfaces.

[6] Marangunić N, Granić A (2015). Technology acceptance model: a literature review from 1986 to 2013. Univ Access Inf Soc.

[7] Mitchell J, Shirota C, Clanchy K (2023). Factors that influence the adoption of rehabilitation technologies: a multi-disciplinary qualitative exploration. J Neuroeng Rehabil.

[8] Smuck M, Odonkor CA, Wilt JK, Schmidt N, Swiernik MA (2021). The emerging clinical role of wearables: factors for successful implementation in healthcare. NPJ Digit Med.

[9] Hill RJ, Fishbein M, Ajzen I (1977). Belief, attitude, intention and behavior: an introduction to theory and research. Contemp Sociol.

[10] Ajzen I, Kuhl J, Beckmann J (1985). Action Control.

[11] Ducey AJ, Coovert MD (2016). Predicting tablet computer use: an extended Technology Acceptance Model for physicians. Health Policy Technol.

[12] Chen SC, Li SH, Li CY (2011). Recent related research in technology acceptance model: a literature review. AJBMR.

[13] Legris P, Ingham J, Collerette P (2003). Why do people use information technology? A critical review of the technology acceptance model. Inf Manag.

[14] Ma Q, Liu L (2005). The technology acceptance model: a meta-analysis of empirical findings. J Organ End User Comput.

[15] King WR, He J (2006). A meta-analysis of the technology acceptance model. Inf Manag.

[16] Rios Rincon AM, Guptill C, Guevara Salamanca JD (2022). Understanding the technology acceptance and usability of a new device for hand therapy: qualitative descriptive study. JMIR Rehabil Assist Technol.

[17] Lee A, Dionicio P, Farcas E (2024). Physical therapists’ acceptance of a wearable, fabric-based sensor system (motion tape) for use in clinical practice: qualitative focus group study. JMIR Hum Factors.

[18] Prill R, Walter M, Królikowska A, Becker R (2021). A systematic review of diagnostic accuracy and clinical applications of wearable movement sensors for knee joint rehabilitation. Sensors (Basel).

[19] Lam AWK, Varona-Marin D, Li Y, Fergenbaum M, Kulić D (2016). Automated rehabilitation system: movement measurement and feedback for patients and physiotherapists in the rehabilitation clinic. Hum-Comput Interact.

[20] Lam AWK, HajYasien A, Kulic D Improving rehabilitation exercise performance through visual guidance.

[21] Qualtrics Qualtrics XM. provo, utah, USA: 2005.

[22] Cabooter E, Weijters B, Geuens M, Vermeir I (2016). Scale format effects on response option interpretation and use. J Bus Res.

[23] DeCastellarnau A (2018). A classification of response scale characteristics that affect data quality: a literature review. Qual Quant.

[24] (2011). Zoom video communications.

[25] Tong A, Sainsbury P, Craig J (2007). Consolidated criteria for reporting qualitative research (COREQ): a 32-item checklist for interviews and focus groups. Int J Qual Health Care.

[26] Argent R, Slevin P, Bevilacqua A, Neligan M, Daly A, Caulfield B (2018). Clinician perceptions of a prototype wearable exercise biofeedback system for orthopaedic rehabilitation: a qualitative exploration. BMJ Open.

[27] Keogh A, Alcock L, Brown P (2023). Acceptability of wearable devices for measuring mobility remotely: observations from the Mobilise-D technical validation study. Digit Health.

[28] Ferguson C, Hickman LD, Turkmani S, Breen P, Gargiulo G, Inglis SC (2021). “Wearables only work on patients that wear them”: Barriers and facilitators to the adoption of wearable cardiac monitoring technologies. Cardiovasc Digit Health J.

[29] Tate JJ, Milner CE (2010). Real-time kinematic, temporospatial, and kinetic biofeedback during gait retraining in patients: a systematic review. Phys Ther.

[30] Owens JG, Rauzi MR, Kittelson A (2020). How new technology is improving physical therapy. Curr Rev Musculoskelet Med.

[31] Huang ZH, Ma CZH, Wang LK, Wang XY, Fu SN, Zheng YP (2022). Real-time visual biofeedback via wearable ultrasound imaging can enhance the muscle contraction training outcome of young adults. J Strength Cond Res.

[32] Bergmann JHM, McGregor AH (2011). Body-worn sensor design: what do patients and clinicians want?. Ann Biomed Eng.

[33] Cajita MI, Hodgson NA, Lam KW, Yoo S, Han HR (2018). Facilitators of and barriers to mHealth adoption in older adults with heart failure. Comput Inform Nurs.

[34] Sprogis SK, Currey J, Considine J (2019). Patient acceptability of wearable vital sign monitoring technologies in the acute care setting: A systematic review. J Clin Nurs.

[35] Jeffs E, Vollam S, Young JD, Horsington L, Lynch B, Watkinson PJ (2016). Wearable monitors for patients following discharge from an intensive care unit: practical lessons learnt from an observational study. J Adv Nurs.

[36] Howard M (2021). Wearables, the marketplace and efficiency in healthcare: how will I know that uou’re thinking of me?. Philos Technol.

[37] Belsi A, Papi E, McGregor AH (2016). Impact of wearable technology on psychosocial factors of osteoarthritis management: a qualitative study. BMJ Open.

[38] Kurtz SM, Higgs GB, Chen Z (2022). Patient perceptions of wearable and smartphone technologies for remote outcome monitoring in patients who have hip osteoarthritis or arthroplasties. J Arthroplasty.

[39] Dunn J, Runge R, Snyder M (2018). Wearables and the medical revolution. Per Med.

[40] Chapman RM, Moschetti WE, Van Citters DW (2021). Is clinically measured knee range of motion after total knee arthroplasty ‘good enough?’: a feasibility study using wearable inertial measurement units to compare knee range of motion captured during physical therapy versus at home. Med Nov Technol Devices.

[41] Díaz S, Stephenson JB, Labrador MA (2019). Use of wearable sensor technology in gait, balance, and range of motion analysis. Appl Sci (Basel).

[42] Hogaboam L, Daim T (2018). Technology adoption potential of medical devices: the case of wearable sensor products for pervasive care in neurosurgery and orthopedics. Health Policy Technol.

